# Study of radiomics based on dual-energy CT for nuclear grading and T-staging in renal clear cell carcinoma

**DOI:** 10.1097/MD.0000000000037288

**Published:** 2024-03-08

**Authors:** Ning Wang, Xue Bing, Yuhan Li, Jian Yao, Zhengjun Dai, Dexin Yu, Aimei Ouyang

**Affiliations:** a Department of Radiology, Central Hospital Affiliated to Shandong First Medical University, Jinan 250013, Shandong Province, P. R. China; b Department of Radiology, Longkou Traditional Chinese Medicine Hospital, Yantai 265700, Shandong Province, P. R. China; c Scientific Research Department, Huiying Medical Technology Co., Ltd, Beijing 100192, P. R. China; d Department of Radiology, Qilu Hospital of Shandong University, Jinan 250012, Shandong Province, P. R. China

**Keywords:** dual-energy CT, nuclear grade, radiomics, renal cell carcinoma, T-stage

## Abstract

**Introduction:**

Clear cell renal cell carcinoma (ccRCC) is the most lethal subtype of renal cell carcinoma with a high invasive potential. Radiomics has attracted much attention in predicting the preoperative T-staging and nuclear grade of ccRCC.

**Objective:**

The objective was to evaluate the efficacy of dual-energy computed tomography (DECT) radiomics in predicting ccRCC grade and T-stage while optimizing the models.

**Methods:**

200 ccRCC patients underwent preoperative DECT scanning and were randomized into training and validation cohorts. Radiomics models based on 70 KeV, 100 KeV, 150 KeV, iodine-based material decomposition images (IMDI), virtual noncontrasted images (VNC), mixed energy images (MEI) and MEI + IMDI were established for grading and T-staging. Receiver operating characteristic analysis and decision curve analysis (DCA) were performed. The area under the curve (AUC) values were compared using Delong test.

**Results:**

For grading, the AUC values of these models ranged from 0.64 to 0.97 during training and from 0.54 to 0.72 during validation. In the validation cohort, the performance of MEI + IMDI model was optimal, with an AUC of 0.72, sensitivity of 0.71, and specificity of 0.70. The AUC value for the 70 KeV model was higher than those for the 100 KeV, 150 KeV, and MEI models. For T-staging, these models achieved AUC values of 0.83 to 1.00 in training and 0.59 to 0.82 in validation. The validation cohort demonstrated AUCs of 0.82 and 0.70, sensitivities of 0.71 and 0.71, and specificities of 0.80 and 0.60 for the MEI + IMDI and IMDI models, respectively. In terms of grading and T-staging, the MEI + IMDI model had the highest AUC in validation, with IMDI coming in second. There were statistically significant differences between the MEI + IMDI model and the 70 KeV, 100 KeV, 150 KeV, MEI, and VNC models in terms of grading (*P* < .05) and staging (*P* ≤ .001). DCA showed that both MEI + IDMI and IDMI models outperformed other models in predicting grade and stage of ccRCC.

**Conclusions:**

DECT radiomics models were helpful in grading and T-staging of ccRCC. The combined model of MEI + IMDI achieved favorable results.

## 1. Introduction

Clear cell renal cell carcinoma (ccRCC) is a type of malignant tumor originating from the urinary system, accounting for about 70%–85% of renal cell carcinoma,^[1]^ and, it is the most lethal subtype with a high invasive potential.^[2]^ The 5-year survival rate of patients with ccRCC is closely related with the pathological nuclear grade.^[3]^ Patients with lower pathological nuclear grade of ccRCC have better prognosis and lower risk of recurrence than those with higher pathological nuclear grade.^[4,5]^ Treatment of RCC includes radical resection, partial resection, tumor enucleation, as well as minimally invasive ablation and targeted therapy developed in recent years. Conservative surgery or minimally invasive ablation can be used for RCC with low pathological grade and staging, and active monitoring or targeted therapy can also be performed in some cases.^[6]^ Tumor T-staging is a comprehensive assessment of tumor progression, and has great significance to the selection of treatment including surgical methods, the formulation of perioperative treatment plan and the prognosis of patients. Biopsy and histopathology are most commonly used for renal cancer grading and staging before operation. However, the disadvantages such as its inherent invasive, hysteresis, in vitro, and dependence on the accuracy puncture tissue limit its application.

Therefore, it is necessary to develop a noninvasive technique for accurately grading the preoperative pathology and T-staging for ccRCC.

Radiomics can extract a large number of image features, combine image quantitative analysis with machine learning, and transform the tumor internal features into rich quantitative features through different algorithms.^[7,8]^ It has shown potential in tumor diagnosis, differential diagnosis, grading, and efficacy evaluation.^[9–13]^ As a noninvasive imaging technology radiomics has attracted much attention in predicting the preoperative T-staging and nuclear grade of ccRCC.

Compared with single energy computed tomography, dual-energy scanning of dual-energy computed tomography (DECT) can obtain mixed energy images (MEI) of different proportions, virtual mono-energy images (VMI), and iodine-based material decomposition images (IMDI) through postprocessing workstation, and significantly improve tissue resolution and material recognition ability.^[14,15]^ Moreover, IMDI can reflect the vascularization of various tissues via measuring the concentration of iodine (contrast reagent)^[16]^ and is conducive to the detection of vascular rich tumor.^[16,17]^

Previous radiomics studies on pathological grading and staging of other tumors based on DECT had obtained good results.^[18–20]^However, the pathological grading and staging of ccRCC based on DECT radiomics are rarely reported. Moreover, there is no consensus with multiple parameters of DECT based radiomics including multiple VMI and IMDI, so it needs to be further studied to find out the best radiomics model. Herein, we investigated the value of radiomics based on the DECT in predicting pathological nuclear grade and T-stage of ccRCC. The efficacies of radiomics models based on different mono-energy VMI, IMDI, and MEI were compared. The potential of DECT as the noninvasive method in clinical decision-making and precision medicine was explored.

## 2. Materials and methods

### 2.1. Patients

This retrospective study was approved by the Institutional Ethics Committee of Jinan Central Hospital Affiliated to Shandong First Medical University and the patient consent was waived. A total of 200 patients with postoperatively pathologically confirmed ccRCC in our hospital from January 2015 to January 2022 were included in the study. There were 137 males and 63 females. Their mean age was 57 ± 11.24 years old and their age range was 33–82 years old. The inclusion criteria were as follows: radical nephrectomy or nephron sparing surgery was performed, and postoperative pathology confirmed ccRCC; complete clinical data could be obtained; and contrast-enhanced DECT of kidney was performed within 1 week before surgery.

The exclusion criteria were as follows: patients with poor image quality that affected the delineation and feature extraction of the region of interest; patients with cardiovascular or renal disease that seriously affected the degree of renal enhancement; patients with previous abdominal surgery; and patients with multiple lesions and poorly defined tumor boundaries.

### 2.2. Pathological staging and nuclear grading

All patients received radical nephrectomy or nephron sparing surgery. Surgical specimens were stained with H&E and examined by 2 pathologists with more than 5 years of professional experience. According to WHO/ISUP nuclear grade of renal cancer, 149 cases were defined as low-grade (grade 1–2), and, 51 cases were defined as high-grade (grade 3–4). According to the AJCC T-staging system, 152 cases had ccRCC at T1–T2 and 48 cases had ccRCC at T3–T4. The final classification and T-staging were decided by the 2 pathologists in consensus. General clinical data of all patients were shown in Table 1.

**Table 1:** General clinical data of all patients of the 200 patients *n* (%).

T-staging						Grading				
	I	II	III	IV	Total	1	2	3	4	Total
Sex										
Male	101 (50.5)	11 (5.5)	14 (7.0)	11 (5.5)	137 (68.5)	9 (4.5)	86 (43.0)	37 (18.5)	5 (2.5)	137 (68.5)
Female	31 (15.5)	9 (4.5)	14 (7.0)	9 (4.5)	63 (31.5)	10 (5.0)	44 (22.0)	7 (3.5)	2 (1.0)	63 (31.5)
Total	132 (66)	20 (10)	28 (14.0)	20 (10)	200 (100)	19 (9.5)	130 (88.22)	44 (22)	7 (3.5)	200 (100)
Age (yr)										
33–60	85 (42.5)	14 (7.0)	21 (10.5)	7 (3.5)	127 (63.5)	16 (8.0)	78 (39.0)	30 (15.0)	1 (0.5)	125 (62.5)
61–82	45 (22.5)	8 (4.0)	7 (3.5)	13 (6.5)	73 (36.5)	2 (1.0)	53 (26.5)	12 (6.0)	8 (4.0)	75 (37.5)
Total	130 (65)	22 (11.0)	28 (14.0)	20 (10.0)	200 (100)	18 (9.0)	131 (66.5)	42 (21.0)	9 (4.5)	200 (100)

### 2.3. DECT imaging acquisition

All patients underwent contrast-enhanced DECT before surgery and signed informed consent before CT scanning. Somatom Force CT scanner (Siemens Healthineers, Forchheim, Germany) was used for scanning. Nonionic contrast agent (Omnipaque, 300 mgI/mL) (1.2 mL/kg; 60–80 mL) was injected intravenously at injection rate of 3.5 mL/s. In dual-energy mode, the cortical phase and parenchymal phase enhanced scanning was performed with the automatic exposure system. The respective parameters were as follows: the delay times were 30 seconds (cortical phase) and 80 seconds (parenchymal phase) respectively; the tube voltages were 100 kvp and sn150 kvp; and the tube currents were 130~180 mAs and 80~90 mAs. The images were reconstructed at 1.0 mm slice thickness and 1.0 mm interval, and then analyzed by using the postprocessing workstation (syngo. via). Finally, the 70 KeV, 100 KeV, 150 KeV, MEI, IMDI, and virtual noncontrasted (VNC) images of the 2 phases were obtained. Then, all these data were imported to radcloud platform (https://mics.huiyihuiying.com/).

### 2.4. Image segmentation and image preprocessing

All images were reviewed and the 3D volume of interests (VOIs) were delineated slice by slice manually by 2 junior radiologists with more than 5 years of working experience in this field, who were blinded to the clinical information of the patients but were aware that the lesions were ccRCC. Then, all contours were reviewed and revised by a senior radiologist with 20 years of experience. If the discrepancy was ≥5%, the tumor borders were determined by the senior radiologist with 20 years of experience.^[21]^ Before VOI segmentation, all images were uniformly enlarged by 1.5 times, and window width and window level were 250/50 HU.

Eventually, the VOIs of 200 patients were segmented on Radcloud platform. The patients were randomized into validation cohort and training cohort at the ratio of about 3:7.

Resampling and filtering were used to reduce noise and increase feature stability. Voxels in each CT image body were resampled to an isotropy voxel size of 1.0 × 1.0 × 1.0 mm^3^ to correct for different voxel spacing and section thickness between different centers. At the same time, the discretization of resampled image data was also used to reduce noise and increase the stability of features. All features were normalized using z-score normalization.

### 2.5. Feature extraction and establishment of the radiomics models

**Figure 1.**
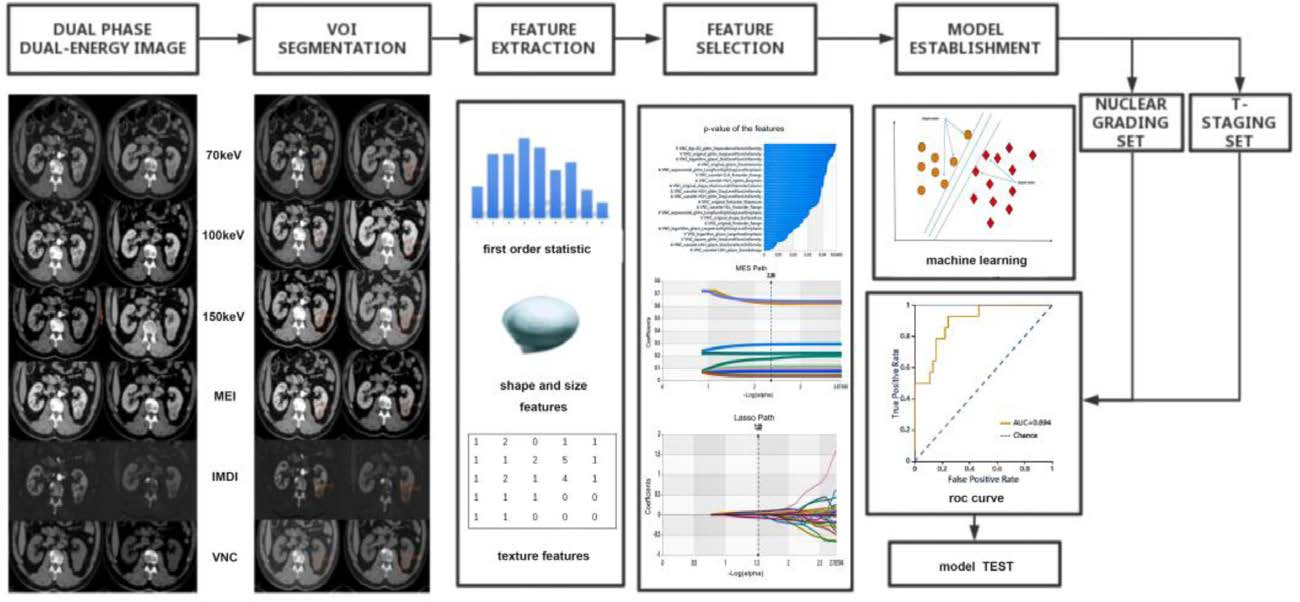
The radiomics analysis workflow. The radiomics workflow includes VOI segmentation, feature extraction, feature selection, model establishment (machine learning, radiomics model), analysis (ROC curve drawing, predictive performance validation and model testing).

The radiomics workflow was shown in Figure 1. A total of 1439 quantitative imaging features were extracted from the VOIs, encompassing 262 first order statistics features delineating the distribution of voxel intensities, 28 3-dimensional features reflecting the shape and size of the region, and 1060 texture features quantifying heterogeneity differences in region characteristics such as gray run length, gray co-occurrence texture matrix (GCTM), gray level size zone matrix, gray level dependence matrix, and neighboring gray tone difference matrix (https://mics.huiyihuiying.com/).

The feature selection methods, including the variance threshold (variance threshold = 0.8), the SelectKBest, and, the least absolute shrinkage and selection operator (LASSO), were used to reduce the redundant features. The optimal features obtained after screening were used for machine learning, and then the classification models were established. Our preexperiments showed that the relative standard deviation of SVM was low and the area under the curve (AUC) was high among KNN, DT, LR and SVM models. Based on the literature^[22,23]^ and our preexperiments, we selected the commonly used support vector machine (SVM) model. The validation method was used to test the effectiveness of the models.

Two groups of models were established according to WHO/ISUP nuclear grading and T-staging. A total of 14 radiomics models were established, including 70 KeV, 100 KeV, 150 KeV, MEI, IMDI, VNC, and MEI + IMDI models of nuclear grading group and T-staging group, respectively.

### 2.6. Qualification and statistical analysis

Feature extraction, dimensionality reduction and modeling were carried out on the Radcloud platform. All statistical analyses were performed by R Studio (version 4.0.2, 2020-06-22) software package. The receiver operating characteristic (ROC) curve was plotted and the area under the ROC curve (AUC) as well as sensitivity and specificity were calculated both in the training cohort and the validation cohort. Delong test was performed to evaluate the differences between the ROC curves. *P* < .05 was considered statistically significant. Decision curve analysis (DCA) was used to assess which model obtained the greatest net benefit.

## 3. Results

### 3.1. Results of nuclear grading group

#### 3.1.1. Dimensionality reduction and selection of task-specific features.

The feature selection methods included the variance threshold (variance threshold = 0.8), SelectKBest, and LASSO in WHO/ISUP nuclear grading group. After reducing the dimensionality, a total of 31 optimal features were selected, including 11 firstorder, 7 GLDM, 2 GLRLM, 9 GLSZM, and 2 shape features. About 7 features were selected from the cortical phase, while 24 features were chosen from the medulla phase. Compared with cortical phase, medullary phase images provided more features to help nuclear classification.

The final number of selected features for the 70 KeV, 100 KeV, and 150 KeV models as well as the MEI, IMDI, VNC, and MEI + IMDI models were determined to be 6, 1, 6, 3, 4, 2, and 5 respectively.

**Figure 2.**
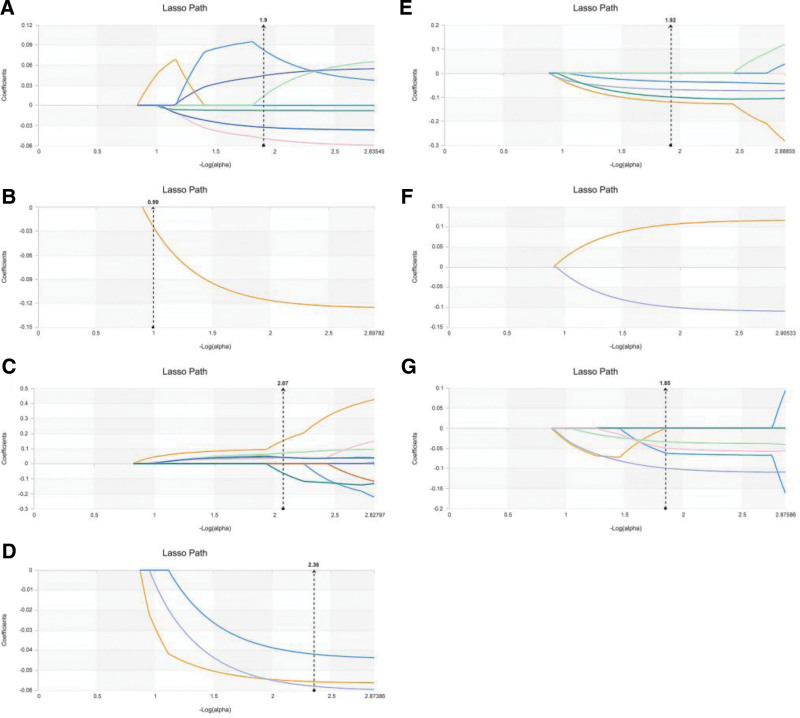
Features extraction and dimensionality reduction for nuclear grading. A–G: LASSO algorithm (regression coefficient diagram) for feature extraction and dimensionality reduction in nuclear grading based on image features at 70 KeV, 100 KeV, 150 KeV, MEI, IMDI, VNC, and MEI + IMDI.

Table 2 and Figure 2 displayed the radiomic features that were selected, along with their corresponding coefficients for each model.

**Table 2:** Description of selected radiomics features with their associated feature group and filter for nuclear grading.

Models for nuclear grading	Radiomic feature	Radiomic class	Filter	Coefficient
70 KeV	Kurtosis	Firstorder	A 70kv_original	0.083
	Kurtosis	Firstorder	A 70kv_exponential	0.014
	LargeDependenceEmphasis	GLDM	V70kv_wavelet-LLH	−0.008
	RunVariance	GLRLM	V70kv_wavelet-LLH	−0.049
	LargeDependenceHighGrayLevelEmphasis	GLDM	V70kv_wavelet-LLH	−0.033
	Kurtosis	Firstorder	V70kv_square	0.044
100 KeV	DependenceVariance	GLDM	V100KV_wavelet-LLH	−0.024
150 KeV	Kurtosis	Firstorder	A150KV_wavelet-LLL	−0.064
	Kurtosis	Firstorder	V150kv_square	0.070
	SmallAreaHighGrayLevelEmphasis	GLSZM	A150KV_wavelet-HHH	0.040
	Maximum3DDiameter	Shape	V150kv_original	0.037
	MinorAxisLength	Shape	V150kv_original	0.041
	Kurtosis	Firstorder	A150KV_logarithm	0.152
MEI	LargeDependenceEmphasis	GLDM	Vmix_wavelet-LLH	0.014
	LargeDependenceHighGrayLevelEmphasis	GLDM	Vmix_wavelet-LLH	0.013
	RunVariance	GLRLM	Vmix_wavelet-LLH	0.038
VNC	HighGrayLevelZoneEmphasis	GLSZM	V VNC.N_wavelet-LLH	0.010
	SmallAreaHighGrayLevelEmphasis	GLSZM	V VNC.N_wavelet-HHH	−0.012
IMDI	RobustMeanAbsoluteDeviation	Firstorder	A VNC_squareroot	−0.040
	DependenceVariance	GLDM	V VNC_wavelet-LLH	−0.070
	MeanAbsoluteDeviation	Firstorder	V VNC_wavelet-LLL	0.000
	HighGrayLevelZoneEmphasis	GLSZM	A VNC_wavelet-HLL	−0.099
MEI + IMDI	MeanAbsoluteDeviation	Firstorder	V VNC_wavelet-LLL	−0.002
	HighGrayLevelZoneEmphasis	GLSZM	A VNC_wavelet-HLL	−0.010
	Variance	Firstorder	V VNC_original	−0.061
	DependenceVariance	GLDM	Vmix_wavelet-LLH	−0.038
	InterquartileRange	Firstorder	V VNC_wavelet-LLL	−0.050

GLDM = Gray Level Dependence Matrix, GLRLM = Gray Level Run Length Matrix, GLSZM = Gray Level Size Zone Matrix

#### 3.1.2. Results of ROC curve analysis and Delong test.

During SVM classifier training, the AUC values for the 70 KeV, 100 KeV, 150 KeV, MEI, IMDI, VNC and MEI + IMDI models were 0.64 (0.66), 0.73 (0.66), 0.97 (0.61), 0.64 (0.63), 0.94 (0.69), 0.77 (0.54), and, 0.89 (0.72) in the training (validation) cohort, respectively.

Among the mono-energy image models, the 70 KeV model exhibited superior classification efficacy. Both the AUC of 70 KeV and IMDI models surpassed that of MEI model. The MEI + IMDI model demonstrated optimal performance in the validation cohort, with an AUC value of 0.72, sensitivity of 0.71 and specificity of 0.70. As anticipated, the VNC model lacked categorical ability.

**Figure 3.**
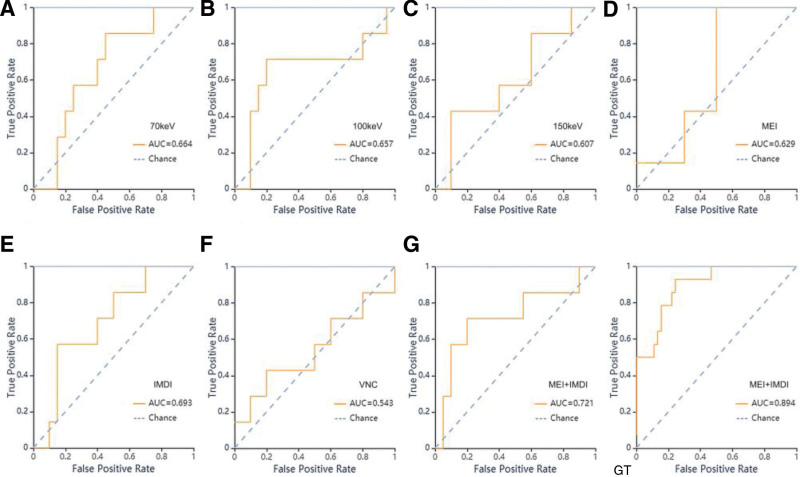
ROC curves of SVM methods for classification in nuclear grading group. A–G: ROC curve of validation set of the 70 KeV, 100 KeV, 150 KeV, MEI, IMDI, VNC and MEI + IMDI models respectively. GT: ROC curve of training set in MEI + IMDI models.

The performance metrics, including AUC value, 95% CI, sensitivity, specificity, and support value of the models developed for nuclear grading in both the training and validation cohorts are presented in Table 3 and Figure 3.

**Table 3:** The results of AUC, 95 CI, sensitivity, specificity for nuclear grading.

	Classifiers	70 KeV	100KeV	150KeV	MEI	IMDI	VNC	MEI + IMDI
Training set	AUC	0.64	0.73	0.97	0.64	0.94	0.77	0.89
	95 CI	0.48–0.80	0.58–0.87	0.87–1.00	0.49–0.80	0.86–1.00	0.62–0.91	0.77–1.00
	Sensitivity	0.60	0.57	0.86	0.57	0.93	0.71	0.79
	Specificity	0.57	0.83	0.93	0.44	0.89	0.72	0.80
	Support	14	14	14	14	14	14	14
Validation set	AUC	0.66	0.66	0.61	0.63	0.69	0.54	0.72
	95 CI	0.44–0.89	0.45–0.86	0.39–0.83	0.41–0.85	0.48–0.91	0.33–0.76	0.52–0.93
	Sensitivity	0.57	0.57	0.43	0.43	0.57	0.43	0.71
	Specificity	0.70	0.85	0.60	0.70	0.80	0.50	0.70
	Support	7	7	7	7	7	7	7

The Delong test demonstrated that the MEI + IMDI model outperformed the 70 KeV, 100 KeV, 150 KeV, MEI and VNC models (*P* < .05) in the validation cohort with statistically significant differences. There were no significant differences in AUC values between the IMDI model and the MEI + IMDI model (Table 4).

**Table 4:** The Delong test of the models’AUC for nuclear grading.

MEI + IMDI vs other 6 models (AUC)	Validation set	*P* value
70 KeV vs MEI + IMDI	0.66 vs 0.72	.033
100 KeV vs MEI + IMDI	0.66 vs 0.72	.008
150 KeV vs MEI + IMDI	0.61 vs 0.72	.001
MEI vs MEI + IMDI	0.63 vs 0.72	.003
IMDI vs MEI + IMDI	0.69 vs 0.72	.128
VNC vs MEI + IMDI	0.54 vs 0.72	<.001

**Figure 4.**
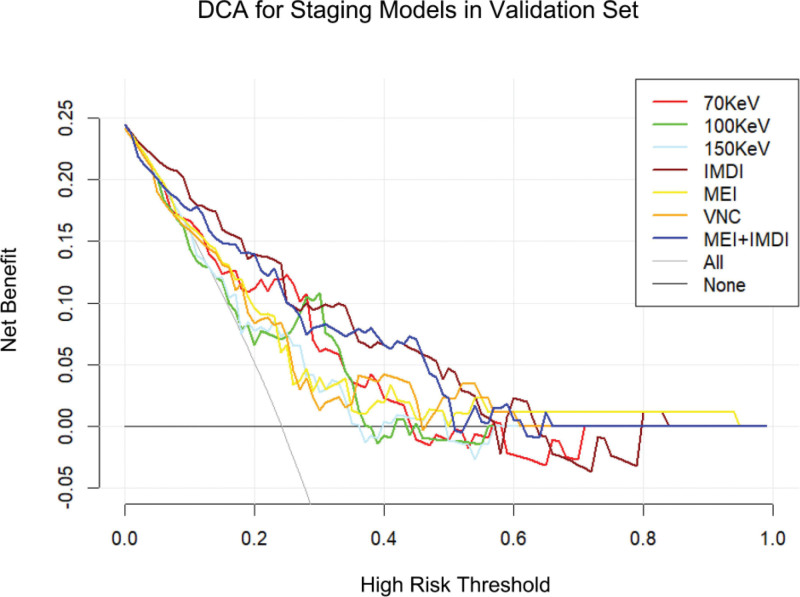
The decision curve analysis of various prediction models for identify high-grade ccRCC from low-grade ccRCC in validation set.

The DCA of the validation group for grading is illustrated in Figure 4. The findings indicate that the MEI + IDMI model enhances the ability to predict nuclear grade of ccRCC at a higher risk threshold, and both MEI + IDMI and IDMI models exhibit superior predictive performance compared to other models in the validation group.

### 3.2. Results of T-stage grouping

#### 3.2.1. Dimensionality reduction and selection of task-specific features.

The feature selection methods included the variance threshold (variance threshold = 0.8), SelectKBest, and the LASSO in T-staging group. After reducing the dimensionality, a total of 56 optimal features were selected for T-staging group, including 18 firstorder, 4 GLDM, 6 GLRLM, 26 GLSZM and 2 shape features. The number of selected features was 28 for both the arterial and venous phases.

The final number of selected features used for the 70 KeV, 100 KeV, 150 KeV, MEI, IMDI, VNC, and MEI + IMDI models were 9, 5, 5, 12, 12, 4, and, 9 respectively.

**Figure 5.**
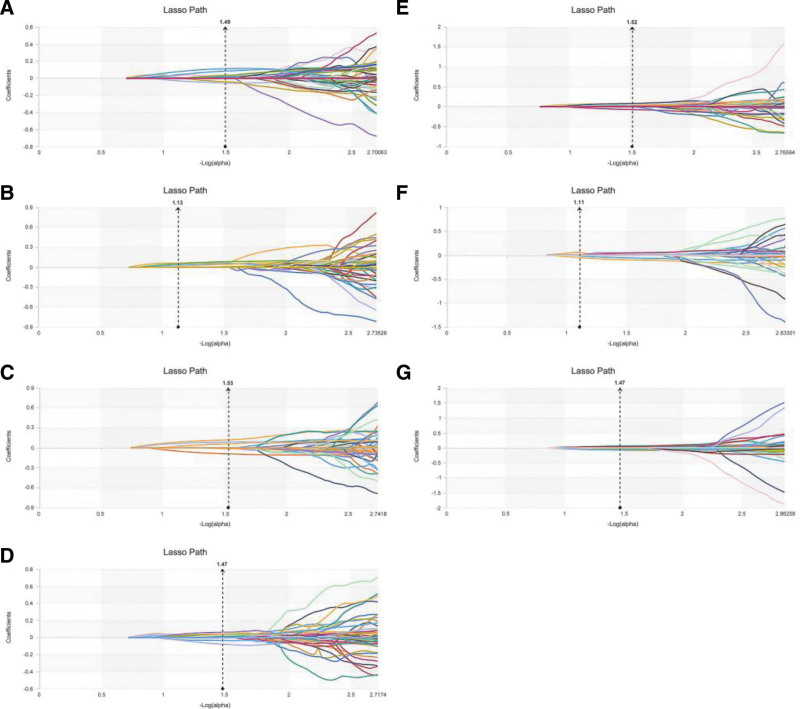
Features extraction and dimensionality reduction for T-staging. A-G: LASSO algorithm (regression coefficient diagram) for feature extraction and dimensionality reduction in T-staging based on image features at 70 KeV, 100 KeV, 150 KeV, MEI, IMDI, VNC, and MEI + IMDI.

The radiomic features selected and their coefficient for each model and the final number of selected features were shown in Table 5 and Figure 5.

**Table 5:** Description of the selected radiomic features with their associated feature group and filter for T-staging.

Models for T-staging	Radiomic feature	Radiomic class	Filter	Coefficient
70 KeV	ZoneEntropy	GLSZM	A 70kv_wavelet-LHH	0.001
	MajorAxisLength	Shape	A 70kv_original	0.017
	GrayLevelNonUniformity	GLSZM	A 70kv_wavelet-HHH	0.032
	ZoneEntropy	GLSZM	A 70kv_wavelet-HLH	0.016
	Skewness	Firstorder	V 70kv_wavelet-HHL	−0.055
	ZoneEntropy	GLSZM	V 70kv_wavelet-LLH	0.112
	Maximum	Firstorder	A 70kv_squareroot	0.034
	HighGrayLevelZoneEmphasis	GLSZM	V70kv_wavelet-HHL	0.084
	DependenceVariance	GLDM	V70kv_wavelet-LLH	−0.041
100 KeV	GrayLevelNonUniformity	GLSZM	A100kv_wavelet-HHH	0.065
	Kurtosis	Firstorder	V 100KV_wavelet-HHL	0.007
	ZoneEntropy	GLSZM	V 100KV_wavelet-LHL	0.054
	SmallAreaHighGrayLevelEmphasis	GLSZM	A 100kv_wavelet-HHH	0.035
	Kurtosis	Firstorder	V 100KV_wavelet-HHH	0.011
150 KeV	Kurtosis	Firstorder	V 150kv_wavelet-HHL	0.120
	ZoneEntropy	GLSZM	A 150KV_wavelet-LLH	0.085
	HighGrayLevelZoneEmphasis	GLSZM	V 150kv_wavelet-LHH	−0.091
	SmallAreaHighGrayLevelEmphasis	GLSZM	A 150KV_wavelet-HHH	0.059
	HighGrayLevelZoneEmphasis	GLSZM	V 150kv_wavelet-HLL	−0.011
MEI	Kurtosis	Firstorder	V mix_wavelet-HHL	0.004
	ZoneEntropy	GLSZM	V mix_wavelet-LHL	0.029
	Skewness	Firstorder	V mix_wavelet-HHL	−0.073
	Kurtosis	Firstorder	V mix_wavelet-HHH	0.030
	MajorAxisLength	Shape	A mix_original	0.060
	ZoneEntropy	GLSZM	V mix_wavelet-HLH	0.002
	ZoneEntropy	GLSZM	V mix_wavelet-HHL	0.029
	ZoneEntropy	GLSZM	V mix_wavelet-HHH	0.003
	LargeDependenceLowGrayLevelEmphasis	GLDM	A mix_wavelet-LHH	0.001
	LargeDependenceLowGrayLevelEmphasis	GLDM	A mix_wavelet-HHH	0.045
	HighGrayLevelZoneEmphasis	GLSZM	V mix_wavelet-LHL	−0.047
	HighGrayLevelZoneEmphasis	GLSZM	A mix_wavelet-HHL	0.028
IDMI	ZoneEntropy	GLSZM	A VNC_wavelet-LHH	0.086
	ZoneEntropy	GLSZM	A VNC_wavelet-HLH	0.023
	RunLengthNonUniformity	GLRLM	A VNC_exponential	0.099
	RunLengthNonUniformity	GLRLM	A VNC_gradient	0.000
	RunLengthNonUniformity	GLRLM	A VNC_square	0.000
	RunLengthNonUniformity	GLRLM	A VNC_lbp-2D	0.000
	Maximum	Firstorder	V VNC_wavelet-LHL	0.070
	Maximum	Firstorder	V VNC_wavelet-HHL	0.020
	Maximum	Firstorder	A VNC_wavelet-LLH	0.013
	Range	Firstorder	V VNC_logarithm	0.023
	HighGrayLevelRunEmphasis	GLRLM	V VNC_wavelet-LLL	−0.078
	Maximum	Firstorder	V VNC_logarithm	0.100
VNC	GrayLevelNonUniformity	GLSZM	A VNC.N_wavelet-LLH	0.061
	HighGrayLevelZoneEmphasis	GLSZM	V VNC.N_wavelet-HHH	−0.049
	Kurtosis	Firstorder	A VNC.N_wavelet-HHH	0.077
	HighGrayLevelZoneEmphasis	GLSZM	A VNC.N_wavelet-HLL	−0.100
	HighGrayLevelZoneEmphasis	GLSZM	A VNC.N_wavelet-HLL	−0.207
MEI + IMDI	HighGrayLevelRunEmphasis	GLRLM	V VNC_wavelet-LLL	−0.048
	SmallAreaHighGrayLevelEmphasis	GLSZM	V mix_wavelet-LHL	−0.078
	TotalEnergy	Firstorder	A VNC_square	0.041
	Maximum	Firstorder	A mix_squareroot	0.039
	ZoneEntropy	GLSZM	A mix_wavelet-HHH	0.060
	Maximum	Firstorder	A VNC_wavelet-LLH	0.043
	Minimum	Firstorder	V VNC_wavelet-LHL	−0.185
	HighGrayLevelEmphasis	GLDM	V VNC_wavelet-LLL	−0.047
	HighGrayLevelZoneEmphasis	GLSZM	V VNC_wavelet-HLL	−0.297

A = Artery phase, GLDM = Gray Level Dependence Matrix, GLRLM = Gray Level Run Length Matrix, GLSZM = Gray-Level Size Zone Matrix, V = venous phase

#### 3.2.2. Results of ROC curve analysis and Delong test.

**Figure 6.**
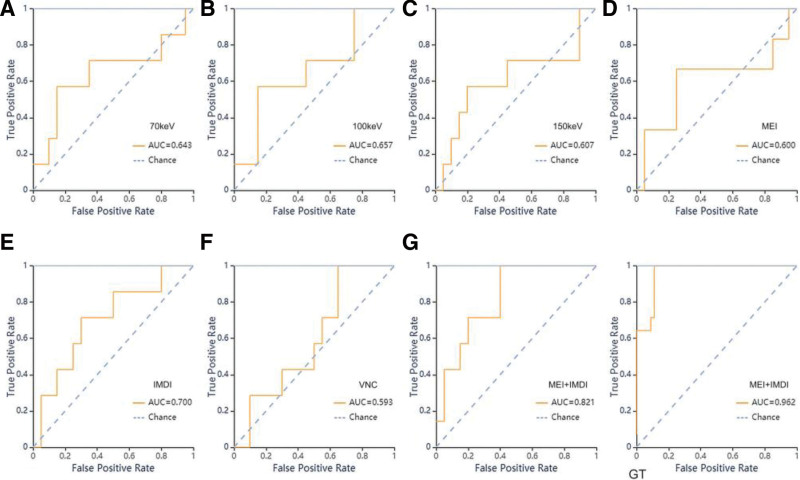
ROC curves of SVM methods for classification in T-staging group. A–G: ROC curve of validation set of the 70 KeV, 100 KeV, 150 KeV, MEI, IMDI, VNC, MEI + IMDI models respectively. GT: ROC curve of training set of the MEI + IMDI model.

When training with SVM classifier, the AUC values of the 70 KeV, 100 KeV, 150 KeV, MEI, IMDI, VNC and MEI + IMDI models were 1 (0.64), 0.87 (0.66), 0.99 (0.61), 0.93 (0.60), 0.87 (0.70), 0.83 (0.593, and 0.96 (0.82) in the training (validation) cohort, respectively (Table 6). ROC curves of SVM methods to classification are shown in Figure 6.

**Table 6:** The results of AUC, 95 CI, sensitivity, specificity for T-staging.

	Classifiers	70 KeV	100 KeV	150 KeV	MEI	IMDI	VNC	MEI + IMDI
Training set	AUC	1.00	0.87	0.99	0.93	0.87	0.83	0.96
	95 CI	0.80–0.97	0.75–0.98	0.91–1.00	0.85–1.00	0.75–1.00	0.70–0.96	0.91–1.00
	Sensitivity	1.00	0.86	0.93	0.93	0.79	0.71	1.00
	Specificity	1.00	0.83	0.91	0.89	0.73	0.78	0.89
	Support	14	14	14	14	14	14	14
Validation set	AUC	0.64	0.66	0.61	0.60	0.70	0.59	0.82
	95 CI	0.49–0.79	0.43–0.88	0.39–0.83	0.37–0.83	0.49–0.91	0.37–0.82	0.62–1.00
	Sensitivity	0.14	0.57	0.57	0.67	0.71	0.43	0.71
	Specificity	0.95	0.65	0.60	0.75	0.60	0.50	0.80
	Support	7	7	7	6	7	7	7

For the models based on different energy images, the AUC value of the MEI was the lowest, but when MEI combined IMDI, the MEI + IMDI model achieved the best performance, with the AUC value 0.96 (0.82) in the training (validation) cohort. The AUC value of 150 KeV model was lower than those of the 70 KeV, 100 KeV models.

As we expected the VNC model had the lowest AUC value among the 7 models.

The AUC, 95% CI, sensitivity, specificity and support value of models for T-staging in the training cohort and the validation cohort are shown in Table 6.

The Delong test compared the predictive performance of the 70 KeV, 100 KeV, 150 KeV, MEI, IMDI, and VNC models with that of the MEI + IMDI model. The results showed that the differences between models have statistical significance (*P* ≤ .001; Table 7).

**Table 7:** The Delong-test of the models’AUC for T-staging.

MEI + IMDI vs other 6 models (AUC)	Validation cohort	*P* value
70 KeV vs MEI + IMDI	0.64 vs 0.82	<.001
100 KeV vs MEI + IMDI	0.66 vs 0.82	<.001
150 KeV vs MEI + IMDI	0.61 vs 0.82	<.001
MEI vs MEI + IMDI	0.60 vs 0.82	<.001
IMDI vs MEI + IMDI	0.7 0 vs 0.82	.001
VNC vs MEI + IMDI	0.50 vs 0.82	<.001

**Figure 7.**
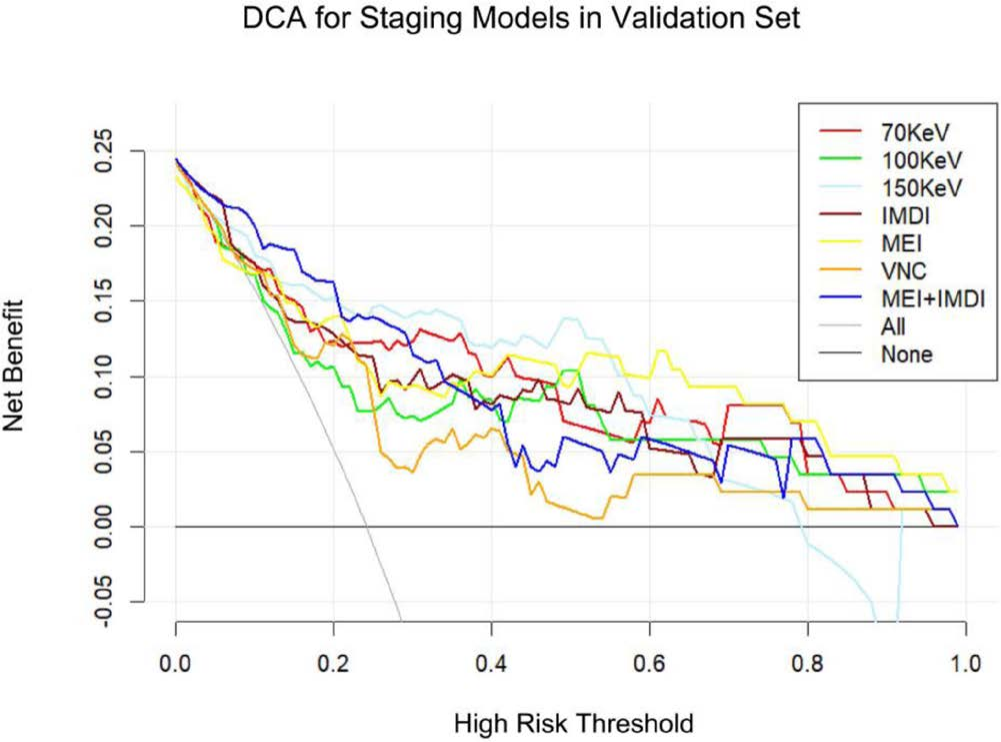
The decision curve analysis of various prediction models for T-stage of ccRCC in validation set.

The DCA of the validation group for T-staging showed that MEI + IDMI model could improve the ability to predict T-stage of ccRCC in a larger risk threshold, and MEI + IDMI and IDMI model in the validation group had higher prediction effect than other prediction models (Fig. 7).

## 4. Discussion

DECT can perform multi-parameter imaging vs single-parameter of conventional CT, and Compared with the hybrid energy images of single energy computed tomography, DECT can obtain a series of VEI and IMDI, moreover, the images can be obtained with a radiation exposure not significantly different from that of single-energy CT.^[24]^ Many studies had applied DECT based radiomics in the tumor staging and nuclear grading and had achieved good results.^[16,19,25,26]^ VMI were recommended for routine use in DECT of the abdomen by the White Paper of the Society of Computed Tomography and Magnetic Resonance^[27]^ and a multi-institutional consensus.^[28,29]^ VMI had been shown to improve lesion detection at low KeV levels.^[24]^ With the increase of energy, the diagnostic accuracy of renal lesions could decrease, 70 KeV VMI has the lowest noise magnitude and may provide the best trade-off between sensitivity and specificity.^[30,31]^Some studies considered 70 KeV VMI accepted as a relatively standard energy level for routine reconstruction.^[24]^

In this study, the radiomics models for nuclear grading and T-staging based on different VMI, IMDI, and MEI images obtained a certain value, The radomics model based on VMI obtained better results than MEI model. The AUC of 70 and 100 KeV models was larger than 150 KeV. This may lend credence to these standpoints.

IMDI can quantify the actual iodine concentration and indicate increased tumor angiogenesis.^[32]^ Homayounieh F et al^[33]^ compared the pathological results of liver lesions with dual-energy IMDI and found that the coincidence rate of IMDI detection results with postoperative pathological examination was higher than that of conventional CT. Wu et al^[34]^ confirmed that radiomics analysis based on IMDI of DECT imaging could provide a relatively high diagnostic value for predicting microsatellite instability status in patients with colorectal cancer. This study showed the combined model of MEI + IMDI for nuclear grading and T-staging in this study achieved better performance in the validation cohort, with the AUC of 0.72 and 0.82, sensitivity of 0.71 and 0.71, and specificity of 0.70 and 0.80, respectively, the AUC of IMDI models were the next highest to combined model of MEI + IMDI. Among the models based on different energy images, the AUC of the MEI was the lowest, but when MEI combined IMDI, the MEI + IMDI model achieved the best performance. IMDI model is expected to play a bigger role in the diagnosis and treatment of ccRCC. This is similar to previous studies.

As we expected, VNC model has the lowest AUC among the models, this is because concentration of iodine can reflect the vascularization of various tissues and provides important information for diagnosis. Without information of iodine VNC provides limited information.

Several studies^[22,23]^ had shown that SVM combined with quantitative MDCT texture analysis has the highest predictive performance in different machine learning based classifiers for distinguishing low-grade from high-grade ccRCC. Our results are similar to theirs, so our research mainly focuses on SVM for machine learning. Generally, radiomics features can be divided into 3 types, including firstorder statistics features, shape- and size-based features and textural features (calculated from gray level run-length and gray level co-occurrence texture matrices).

In our study, among all selected radiomics features, the number of texture features was the highest, with the number 1060/1439. The texture features showed higher discrimination ability. The reason for the good performance is that 3D texture features can provide the overall characteristics of tumor heterogeneity by analyzing the gray distribution of pixels or pixels in CT images and its relationship with gray level.^[35]^ Radiomics is mainly composed of 3D texture features, and its prediction performance is significantly superior to morphological features and firstorder features.^[36,37]^ Mayerhoefer et al^[38]^ showed that radiomics could be used to describe tumor heterogeneity. According to previous studies, the risk of malignancy in high-grade tumors can increase with tumor size, and tumor size is significantly correlated with metastasis.^[39]^ Shape features refer to the characteristics that describe the size and morphology of a region of interest, such as maximum 2-dimensional diameter, volume, and area. These parameters reflect information about the entire tumor shape. Our findings are consistent with this conclusion. Our study also has some limitations. Firstly, the sample size was relatively small and cases were not evenly distributed across different grades or stages. Secondly, the T-staging subgroups (T1–2 and T3–4) were coarse due to clinicians’ emphasis on other subgroups of T-staging (such as T1a and T1b). Finally, this study is limited to a single center and lacks external validation. In the future, multi-center studies should be carried out to enhance the generalizability of the model.

## 5. Conclusion

Radiomics models based on DECT have the potential to aid in nuclear grading and T-staging of ccRCC prior to surgery, thereby facilitating treatment strategies and prognosis assessment. This provides additional incremental value for the development and utilization of DECT.

## Author Contributions

**Writing—original draft:** Ning Wang, Xue Bing.

**Writing—review & editing:** Ning Wang.

**Data curation:** Xue Bing, Yuhan Li.

**Funding acquisition:** Jian Yao, Aimei Ouyang.

**Methodology:** Jian Yao, Dexin Yu.

**Software:** Zhengjun Dai.

**Project administration:** Aimei Ouyang.
